# Ethanol extract of the mushroom *Coprinus comatus* exhibits antidiabetic and antioxidant activities in streptozotocin-induced diabetic rats

**DOI:** 10.1080/13880209.2022.2074054

**Published:** 2022-06-08

**Authors:** Nuniek Ina Ratnaningtyas, Hernayanti Hernayanti, Nuraeni Ekowati, Fajar Husen

**Affiliations:** a Department of Biology, Faculty of Biology, Jenderal Soedirman University, Central Java, Indonesia; b Department of Medical Laboratory of Technology, Bina Cipta Husada College of Health Science, Central Java, Indonesia

**Keywords:** Bioactive compounds, diabetes mellitus, DPP-4, GLP-1, reactive oxygen species

## Abstract

**Context:**

Edible mushrooms have a long history of use in traditional Chinese or Japanese medicine. *Coprinus comatus* (O.F. Müll.) Pers. (Agaricaceae) contains antioxidant and antidiabetic agents.

**Objective:**

To identify the benefits of ethanol extracts of the *C. comatus* fruit body in streptozotocin-induced hyperglycaemic rats by evaluating their blood glucose, glycosylated haemoglobin (HbA1c), insulin, glucagon-like peptide-1 (GLP-1), dipeptidyl peptidase-4 (DPP-4), and glutathione (GSH) levels, with and without extract administration.

**Materials and methods:**

Wistar rats were either left untreated or were administered 45 mg/kg body weight (BW) streptozotocin; 45 mg/kg BW metformin; or 250, 500, or 750 mg/kg BW extract for 14 days. The blood glucose, GLP-1, DPP-4, GSH, insulin, and HbA1c levels were determined. Data were analysed using analysis of variance and Duncan’s multiple range tests.

**Results:**

Preliminary data showed that administration of *C. comatus* ethanol extract dose of 250, 500, and 750 mg orally has no toxicity effects after 24 h administration. The ethanolic extract of fruiting body of *C. comatus* considerably reduced the rat’s fasting blood glucose levels 26.69%, and DPP-4 6.97% at dose of 750 mg. The extract reduced HbA1c 4–4.30%, increased GLP-1 71.09%, GSH 11.19% at dose of 500 mg, and increased insulin levels 13.83%. Extracts contain bioactive compounds such as flavonoid, alkaloid, terpenoids, vitamins C and E, rutin, and saponin.

**Conclusions:**

The *C. comatus* extract can be used as herbal medicine that reduces diabetic symptoms. Further investigation on *C. comatus* extracts should be conducted with gas chromatography-mass spectrometry to characterise the bioactive compounds.

## Introduction

Diabetes mellitus (DM) is a metabolic disease associated with disordered carbohydrate metabolism and decreased or absence of insulin sensitivity and production. Diabetes mellitus is strongly associated with several diseases, including cardiovascular complications, heart attacks, and obesity, resulting in microvascular complications such as blood vessel sclerosis, which can progress to myocardial infarction (Sabo et al. 2010). The metabolic abnormalities associated with diabetes may result in an oxidative stress reaction in the pancreatic cell, adversely affecting insulin activity via multiple interacting pathways. Numerous signalling pathways in cells, for example, NF-κB (nuclear factor-κB) and PKC (protein kinase C), may interfere with insulin signalling pathways, resulting in the development of insulin resistance in type 2 diabetes patients; additionally, it may activate ROS (reactive oxygen species) and generate ROS such as hydrogen peroxide and superoxide anions. These species may deteriorate the pancreas’s Islets-cells, resulting in the decreased insulin release seen in diabetes mellitus (Ma et al. 2018).

According to the International Diabetes Federation (IDF) in 2019, 463 million adults aged 20–79 years worldwide had diabetes, including type 1 and type 2 diabetes, both diagnosed and undiagnosed. 79.4% of sufferers were estimated to live in low- and middle-income countries. As shown by 2019 estimations, 578.4 million adults are predicted to have diabetes by 2030, and 700.2 million adults will have diabetes by 2045. Indonesia is ranked seventh in 2019, with an average of 10.7 million adults who have diabetes, which is expected to rise to 13.7 million by 2030 and 16.6 million by 2045 (International Diabetes Federation [IDF] 2019). However, a more recent study estimated that there are 232 million people who are undiagnosed with diabetes (Song et al. 2021).

Currently, synthetic drugs for DM treatment are widely available, yet synthetic drugs come with adverse side effects (American Diabetes Association [ADA] 2017). Instead of single-target traditional drugs, multi-component and multi-target therapies that combine traditional drugs and herbal medicine have been shown to have lesser side effects and are more effective than single-component treatments. Indeed, an increasing number of studies and rapid development of natural products with anti-diabetic properties have bolstered the case for using these types of combinational therapies (Li et al. 2017). Additionally, it is estimated that 80% of the world’s diabetic population currently relies on herbal medicine for their ongoing treatment (Oloyede et al. 2015).

*Coprinus comatus* (O.F. Müll.) Pers. Agaricaceae has been used as an antidiabetic food for centuries, particularly in countries such as China; its bioactive compounds, which include polysaccharides, proteins, alkaloids, terpenoids, sterols, and phenolics, have been shown to have a variety of health benefits (Gulati et al. 2019). *Coprinus comatus*, a macroscopic mushroom, has grown in popularity as a food in China; 382,000 tons of *C. comatus* were produced in 2006, owing to its flavourful taste, nutritional value, and shape resembling a chicken drumstick, which has earned it the nickname ‘chicken drumstick mushroom’ (Li et al. 2010).

The bioactive compound in *C. comatus* can lower blood glucose levels (Yamac et al. 2009). Certain natural medicines, such as ethanol extracts of *C. comatus*, have significant potential as alternative herbal medicines due to their ability to improve insulin sensitivity and release in peripheral tissue; additionally, they have fewer adverse effects than synthetic medications, which are frequently toxic when used long-term (Hwang et al. 2005; Baldeón et al. 2012). In this study, it was found that administering ethanol extracts from the fruiting body of *C. comatus* at a dose of 1000 mg/kg to alloxan-induced diabetic rats increased insulin levels by 10.50 mIU/mL, compared to the NC group that received no treatment, implying that this extract may optimise insulin production (Ratnaningtyas et al. 2019).

The administration of *C. comatus* extracts has great potential for protecting pancreatic β-cell against free radical damage since they can act as antioxidants that increase superoxide dismutase (SOD) levels (Yu et al. 2009; Ratnaningtyas et al. 2019). The *C. comatus* ethanol extracts from fruit bodies, mycelia extracts, and fermentation filtrate of mycelia can scavenge 84.5% of 2,2-diphenyl-1-picrylhydrazyl (DPPH) radicals at 5 g/mL, 48.4% at 10 mg/mL, and 51.2% at 20 mg/mL (Tsai et al. 2009).

Glucagon-like peptide-1 (GLP-1) is a gastrointestinal hormone that plays a critical role in the intestine’s glucose metabolism and acts as an insulinotropic hormone (i.e., stimulator of insulin secretion) (Zhang et al. 2019). GLP-1’s effectiveness and levels decline to suboptimal levels in DM due to inhibition by the enzyme dipeptidyl peptidase-4 (DPP-4), which rapidly degrades GLP-1, resulting in its very short lifespan. The bioactive alkaloids in *C. comatus* function as DPP-4 inhibitors by enhancing the uninterrupted flow of GLP-1 and accelerating phosphatidylinositol (PI) 3-kinase (PI-3K) activity, which enhances insulin biosynthesis and cell proliferation (Drucker 2007; Shukla and Srinivasan 2012). Previous research confirmed that the ethyl acetate extract of *C. comatus* 500 mg was effective in increasing levels of GLP-1 (Husen et al. 2021).

This study was conducted to analyse the potential effects of *C. comatus* ethanol extracts. Blood glucose, glycosylated haemoglobin (HbA1c), insulin, GLP-1, DPP-4, and glutathione (GSH) levels were evaluated in a streptozocin (STZ)-induced hyperglycaemic rat model administered *C. comatus* ethanol extracts.

## Materials and methods

### Mushrooms and animals

The native specimen of *C. comatus* mushroom was collected from CV. Asa Agro Corporation in 2019, October from Cianjur in the form of pure culture and fruit bodies. N I Ratnaningtyas and T U Priyadi authenticated and identified the mushroom materials (voucher specimen unavailable). Pure cultures were stored and maintained on potato dextrose agar slants at 3 °C and subcultured periodically, while the fruit body of the mushroom was cut into small pieces and dried in the oven at 45 °C. The stock of fruit body pieces was stored at 6–8 °C. Male Wistar rats were obtained from UD. Wistar, Bantul, Yogyakarta, Indonesia. Metformin (GEN DEXA 500MG of PT. Dexa Medica, Cikarang, Indonesia) at 45 mg/kg BW (based on a dose conversion from human to rat) (Katzung 2012) as a positive control.

### Preparation of *C. comatus* ethanol extract

The *C. comatus* fruit body (1.5 kg) was cut into small pieces and dried in an oven at 40–45 °C. The dried fruit body was blended to obtain a mushroom powder. Mushroom powder (500 g) was macerated in ethanol pro-analysis EMPARTA^®^ ACS solvent used maceration method with 3 repetitions and 3 ratio mushroom powder-solvent to obtain more macerate of mushroom. On day 1, 500 g mushroom powder was macerated with 2500 mL ethanol (1:5 ratio), on day 2 the 500 g mushroom powder was re-macerated with 1500 mL ethanol (1:3) and on day 3 was re-macerated with 1000 mL of ethanol (1:2 ratio). Total of macerate from days 1–3 was collected and vacuum-filtered (Millipore Rotavapor^®^), pooled, and evaporated using a vacuum rotary evaporator (Olympus OLS5100) until a thick extract was obtained (Widyastuti et al. 2013). The extract was stored at 8 °C in a refrigerator before used for treatment. Our preliminary data also showed that individual administration of *C. comatus* ethanol extract dose of 250, 500, and 750 mg orally has no toxic effects, no systemic side effects or death in animals after 24 h compared to control rats which received distilled water, all rats are alive and healthy. Therefore, these doses were used for the treatment.

### Animal treatment and housing conditions

The experimental design and procedures were approved by the Health Research Ethics Committee of Dr. Moewardi General Hospital (Surakarta, Central Java, Indonesia; ethical approval number: 297/II/HREC/2020) based on the ethical principles of replacement, reduction, and refinement (3 R); the freedom from hunger and thirst, freedom from discomfort, freedom from pain, injury and disease; the freedom from fear and distress; and the freedom to express natural behaviour (5 F).

This study used 30 male Wistar rats (150–200 g) that were housed in polycarbate cages (5 rats for each cage) covered with perforated wire in a climate-controlled room (temperature, 23 ± 2 °C; humidity, 55 ± 5%), and were fed biopellets (CitraFeed of Citra Ina Feedmill PT.) twice a day, in the morning (08:00) and evening (17:00), and were provided *ad libitum* access to distilled water. The rat cages were cleaned every 3 days by replacing the base of the cage. Before treatment, all rats were confirmed to be in good physical health.

### STZ induction procedure

STZ was diluted in 2.5 mL 0.5 M citrate buffer (pH 4.5) to obtain a dose concentration of 45 mg/kg BW. This solution was intraperitoneally administered to the rats (Abeeleh et al. 2009).

The animals were acclimated for 14 days before treatment and housed in cages containing woodchip bedding under standard temperature and humidity conditions (temperature: 22–24 °C; relative humidity: 50–60%), in a pathogen-free room on a 10 h light/dark cycle (lights on at 07:00) with free access to food and water to ensure that they had the appropriate BW and were in good physical condition for STZ induction. Before treatment was initiated, rat BW was measured to obtain baseline levels. The rats with the glucose levels 180–250 mg/dL in fasting conditions were confirmed and categorised as hyperglycaemia rats. The rats in this study were randomly divided into six groups using a simple randomising technique; each group was composed of 6 rats with one used for drop out estimation total of 36 rats were used (Pratiwi et al. 2018). The groups were:

HC: healthy control (no STZ induction; no extract administered)
NC: negative control (45 mg/kg BW STZ induction; no extract administered)
PC: positive control (STZ induction and administered 45 mg/kg BW metformin)
T1: treatment group 1 (STZ induction and administered 250 mg/kg BW extract)
T2: treatment group 2 (STZ induction and administered 500 mg/kg BW extract)
T3: treatment group 3 (STZ induction and administered 750 mg/kg BW extract)

The *C. comatus* ethanol extract was orally administered using a stomach sonde needle 2 h before feeding. The extracts were administered once a day in the morning for 14 days.

### Blood sample collections

Before blood samples were collected all rats fasted for 10–12 h. Initial blood glucose levels were done by tail snip of the rats and allowing blood to drop on the glucometer strip, and the value was read off on the screen of the glucometer that used to determine the hyperglycaemic rats. On day 15, blood samples were taken which was conducted through orbital venous plexus used tube capillary and sacrificed with 20 mg/kg BW diethyl ether anaesthesia by inhalation to reduce pain before blood was draw follow the ethical approval and procedure (approval number: 297/II/HREC/2020). Samples were collected in EDTA tube (Vaculab EDTA K3 3 mL/3 cc @OneMed). Plasma samples were obtained through centrifugation method at 3000 rpm for 5 min (Centrifuge Gemmy PLC-05 1000-4500 rpm)

### Bioactive compound identification

Flavonoids were examined by taking 2 mL mushroom extracts and steaming it for 5 min, after which 0.1 mL of HCl was added. Samples with flavonoids showed a colour change from yellow (+) or orange (++) to red (+++) (Ergina and Pursitasari 2014). For the polyphenol test, 5 mL distilled water was added to each sample then steamed for approximately 5 min; 2 drops FeCl_3_ were added, and colour changes were observed. The colours yellow (+), brown (++), and dark brown (+++) indicated a positive result for polyphenols. For the terpenoid test, 3 drops HCl and 1 drop 0.05 M H_2_SO_4_ were added to 2 mL extract. Appearance of a green colour indicated the presence of terpenoids in the sample. For the detection of saponins, distilled water was boiled, followed by the addition of 2 mL methanol. The sample was cooled and mixed with shaking for approximately 10 s. The formation of a stable foam indicated the presence of saponins (Shah et al. 2018). Spectrophotometric analysis of flavonoids and total alkaloids was performed at 510 nm (Suresh et al. 2020). HPLC-based qualitative and quantitative analyses were performed using a C-18 reverse-phase column (Develosil^®^ ODS-UG-3) with an HPLC-Shimadzu. The mobile phase consisted of 25% acetonitrile in 0.025 M KH_2_PO_4_ (Meyer 2010).

### Analysis of main diabetic parameters

The main diabetic parameters were analysed using serum samples obtained on day 15 from all groups. Blood glucose levels were measured using a glucometer (Gluco.Dr, All Medicus Co., Ltd., Gyeonggi-do, Republic of Korea). HbA1c levels were measured using the A1C EZ 2.0 Glycohemoglobin Analyser from BioHermes (Wuxi BioHermes Biomedical Technology Co., Ltd., Jiangsu, China). GSH was measured using a glutathione reductase assay kit from Randox Laboratories Ltd., Crumlin, UK (Cat.No GR2368). Rat-specific DPP-4 (Cat.No E0226Ra), insulin (Cat.No E0707Ra), and GLP-1 (Cat.No E0719Ra) ELISA kits (BT Laboratories, Shanghai, China) were used for the measurement of the corresponding parameters (BT Laboratory 2018).

### Histological preparations

A 5 mm piece of pancreatic tissue was fixed in neutral buffered formalin (10%) for at least 24 h and processed for light microscopy. Each piece of the rat pancreas paraffin block was sectioned (7 µ) before staining with haematoxylin and eosin (H&E). Then micrographs of the slides were taken using an Olympus BX50 microscope fitted with an Olympus DP70 digital camera. Each digital micrograph was studied for surface area and number of cells of Langerhans Islets using an image-analysing program (Soft Imaging System LS in Olympus Com. v. 5.0) (Yamac et al. 2009; Sabo et al. 2010).

### Statistical analysis

Data are shown as mean ± standard error (SE) and were analysed using one-way analysis of variance (ANOVA) and Duncan’s multiple range tests. The IBM SPSS for Windows version 20.0 (IBM Corp., Armonk, N.Y., USA) was used to compare the main parameters of the six study groups. A *p* ≤ 0.05 was considered to indicate statistical significance.

## Results

The extraction procedure of 500 g *C. comatus* mushroom dried simplicia and ethanol pro-analysis grade solvent, 2000 mL yielded 12 g *C. comatus* dense extract. Hyperinsulinemia effect was present in 1000 mg dose with insulin levels of >10 mIU/mL in fasting condition, to minimise this effect the dose is reduced. Meanwhile, the lowest dose of 250 mg is chosen to determine the lower dose limit that can have antidiabetic and antioxidant effect.

**Figure 1.**
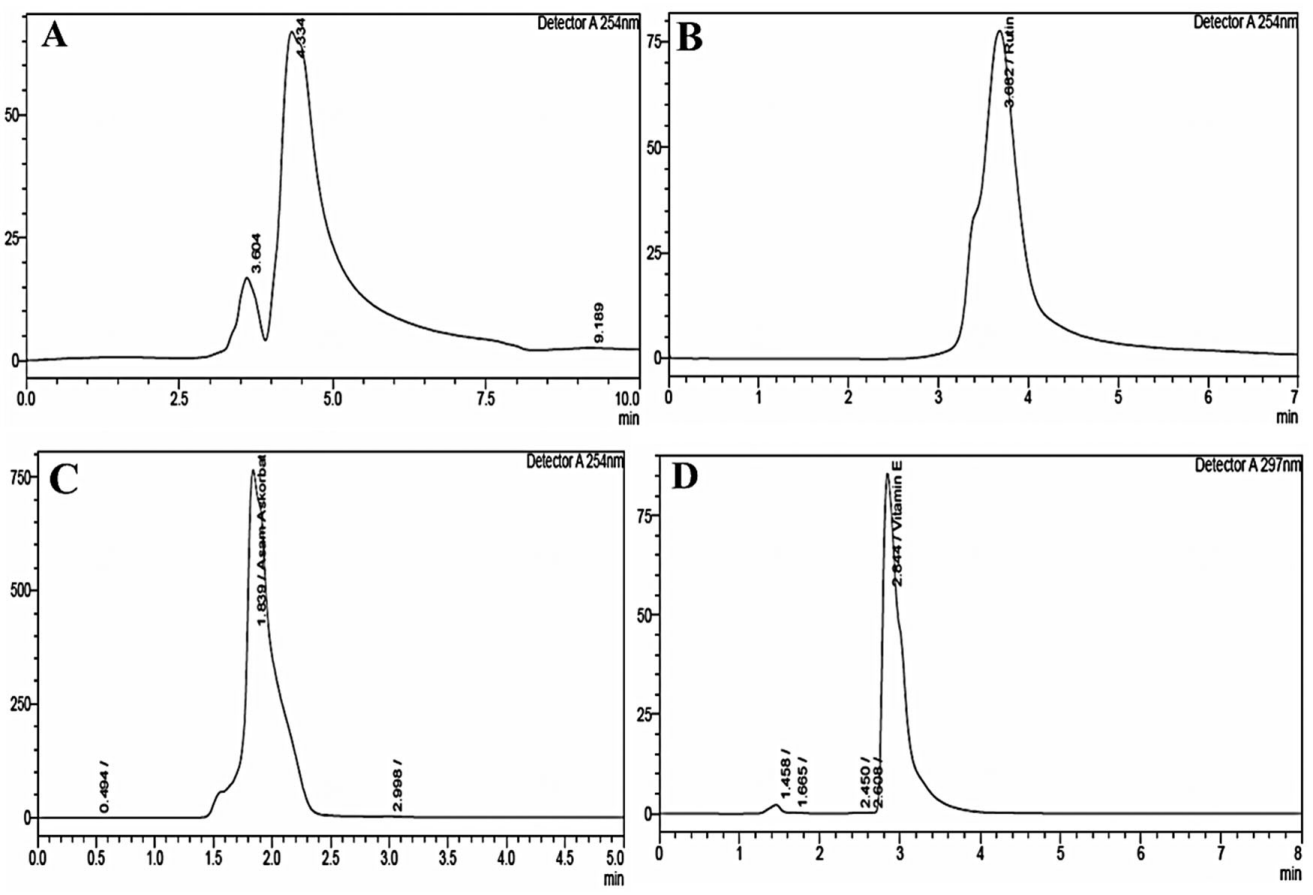
Chromatograms standard of quercetin, rutin, vitamin C and vitamin E. Chromatograms of (A) standard of quercetin; (B) standar of rutin; (C) ascorbic acid (vitamin C); (D) vitamin E (see Supplementary material 1).

**Figure 2.**
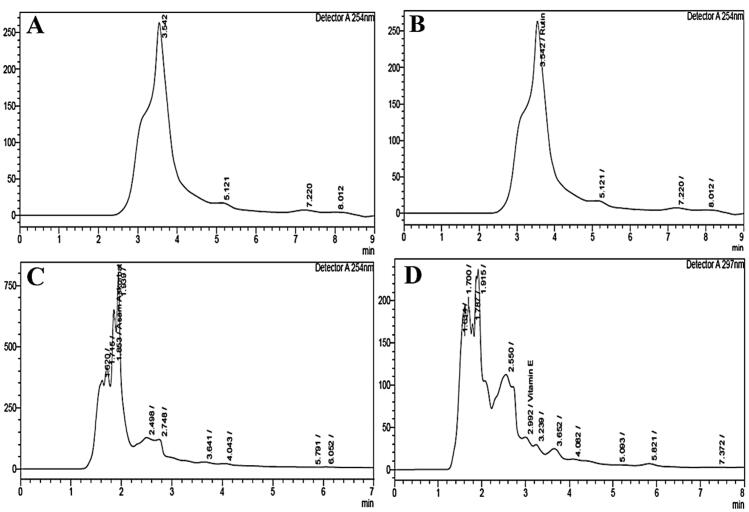
Chromatographic sample of quercetin, rutin, vitamin C and vitamin E. Chromatographic sample of *C. comatus extract* detects (A) quercetin; (B) rutin; (C) ascorbic acid (vitamin C); (D) vitamin E (see Supplementary material 2).

Qualitative and quantitative spectrophotometry (Table 1) and high-performance liquid chromatography (HPLC) were used to analyse phytochemicals and bioactive compounds in the *C. comatus* ethanol extract and showed the presence of rutin, vitamins C and E (Table 2). The chromatogram result of HPLC analysis of standard and sample of *C. comatus* extract are shown in Figure 1 (chromatogram standard) and Figure 2 (chromatogram sample).

**Table 1.** Qualitative analysis of *C. comatus* and spectrophotometer result.

Phytochemical/Bioactive compound	Reagent	Result (qualitative)	Result (quantitative) spectrophotometer
Flavonoid	Mg/Zn + HCl + Amyl Alcohol	Reddish yellow (++)	16.96 mg/L (32.8%)
Alkaloid	Dragendorff dye	Dark orange (+)	3.84 mg/L (7.69%)
Saponin	Aquades	Bubble formed (+)	–

*Note*: qualitative range/level; + (low), ++ (medium), +++(high), – (not quantitatively analysed).

**Table 2.** Bioactive compound of *C. comatus* ethanol extract through HPLC analysis.

Bioactive compounds target	Detected compounds	Retention time	Concentration
Flavonoid (quercetin and rutin)	Rutin	3.163	495.608 ppm
	Quercetin (ND)	–	–
Vitamin C (ascorbic acid)	Ascorbic acid	1.853	152.106 mg/L
Vitamin E (α-tocopherol)	α-Tocopherol	2.992	53.856 g/L

*Note*: Retention time: standard flavonoids (quercetin): 4.334 (100 ppm), flavonoids (rutin): 3.682 (100 ppm), Vitamin C (ascorbic acid): 1.839 (500 mg/L), Vitamin E (α-tocopherol): 2.844 (150 g/L). ND: not detected.

HPLC analysis using standard quercetin and rutin at 100 ppm concentration (Figure 1) with a wavelength of 254 nm in the sample extract of *C. comatus* did not detect the presence of quercetin but detected rutin (Figure 2). Meanwhile, the standard vitamin C using ascorbic acid at 500 mg/L concentration (Figure 1) detects vitamin C at a concentration of 152,106 mg/L (Figure 2). Vitamin E was detected at 53,856 g/L with a standard concentration of 150 g/L (Figure 1).

This research only used male Wistar rats due to the characteristics of the female counterpart that are less susceptible to streptozotocin (Etuk 2010). Male Wistar rats have hormonal conditions that are more stable whereas female rat’s hormonal conditions can be influenced by the oestrus cycle (Shulman and Spritzer 2014) which may affect the results of the study. The samples used for the analysis of blood glucose, plasma insulin, GLP-1, HbA1c, and GSH levels were obtained from blood serums after 14 days of extract treatment. The average blood glucose levels (*p* < 0.05) showed significantly different after induction measured under fasting conditions was 149.02–175 mg/dL (Table 3), which is an early indication of hyperglycaemia (Ratnaningtyas et al. 2019). The mean initial blood glucose levels for all STZ-induced treatment groups ranged from 149 to 175 mg/dL, while the mean final blood glucose levels were between 110 and 270 mg/dL (Table 3).

**Table 3.** Average and decreasing percentage of initial and late blood glucose levels.

Treatment group	Initial blood glucose (mg/dL)	Late blood glucose (mg/dL)	Decreasing percentage (%)
HC	101.25 ± 4.500^a^	112.5 ± 8.660^a^	11.11 ± 13.645^a^
NC/IC	162.5 ± 14.341^b^	269.25 ± 121.373^b^	65.69 ± 61.326^b^
PC	175 ± 44.929^b^	127 ± 25.806^a^	29.14 ± 19.833^a^
T1	164.75 ± 35.929^b^	149.75 ± 32.867^a^	9.71 ± 8.742^a^
T2	149.02 ± 7.659^b^	130.5 ± 3.872^a^	12.43 ± 6.829^a^
T3	150.3975 ± 21.748^b^	110.25 ± 11.026^a^	26.69 ± 7.840^a^

*Note*: Numbers on each line followed by the same letter are not significantly different, values are expressed as mean ± SD (*n*: 24) *p* < 0.05. HC: Healthy control; NC: negative control (45 mg STZ); PC: positive control (45 mg metformin); T1: 250 mg ethanol extract of *C. comatus*; T2: 500 mg ethanol extract of *C. comatus*; T3: 750 mg ethanol extract of *C. comatus*.

After administration of 750 mg/kg BW of *C. comatus* ethanol extract for 14 days (Group T3), we found that the blood glucose level showed a significant decrease (26.69%), while that of the negative control (NC) group (induced by STZ) increased by 65.69% and presented with chronic hyperglycaemia.

The NC rat group had an HbA1c level (*p* < 0.05) showed significantly different, which was greater than the normal limit of the HC rat group, measured in fasting conditions (3.5–4%). Rats administered 500 mg/kg BW extract had the lowest HbA1c level (4.075%) of the treatments tested, which was close to normal conditions (Table 4).

**Table 4.** Average of glycated haemoglobin (HbA1c) levels.

Treatment group	HbA1c levels (%)
HC	3.55 ± 0.472^a^
NC/IC	4.7 ± 0.804^c^
PC	4.275 ± 0.991^ab^
T1	4.35 ± 0.173^ab^
T2	4.075 ± 0.550^ab^
T3	4.1 ± 0.270^ab^

*Note*: Numbers on each line followed by the same letter are not significantly different, values are expressed as mean ± SD (*n*: 24) *p* < 0.05. HC: Healthy control; NC: negative control (45 mg STZ); PC: positive control (45 mg metformin); T1: 250 mg ethanol extract of *C. comatus*; T2: 500 mg ethanol extract of *C. comatus*; T3: 750 mg ethanol extract of *C. comatus*.

**Figure 3.**
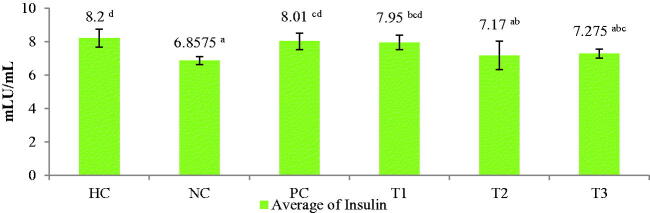
Insulin hormone levels after extract treatment. Histograms with the same letter are not significantly different, values are expressed as mean ± SD (*n*: 24) *p* < 0.05. HC: Healthy control; NC: negative control (45 mg STZ); PC: positive control (45 mg metformin); T1: 250 mg ethanol extract of *C. comatus*; T2: 500 mg ethanol extract of *C. comatus*; T3: 750 mg ethanol extract of *C. comatus*.

The experimental groups treated with the *C. comatus* ethanol extract showed significantly different insulin levels than the NC group (*p* < 0.05). The NC group had an average insulin level <7 mIU/mL (6.85 mIU/mL), while the experimental groups that were administered different doses of the extract all showed levels above 7 mIU/mL (Figure 3).

**Figure 4.**
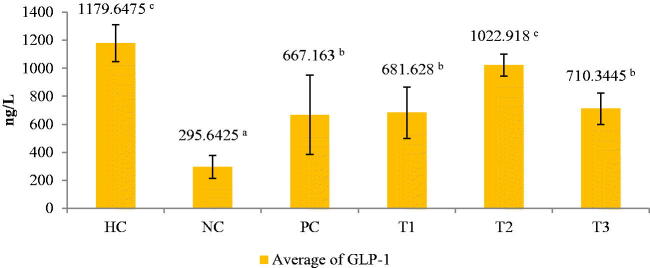
Glucagon-like peptide (GLP)-1 levels after extract treatment. Histograms with the same letter are not significantly different, values are expressed as mean ± SD (*n*: 24) *p* < 0.05. HC: Healthy control; NC: negative control (45 mg STZ); PC: positive control (45 mg metformin); T1: 250 mg ethanol extract of *C. comatus*; T2: 500 mg ethanol extract of *C. comatus*; T3: 750 mg ethanol extract of *C. comatus*.

The GLP-1 level significantly different in the NC group that was not administered the *C. comatus* ethanol extract was very low (29.642 ng/L, below the normal level) (*p* < 0.05), whereas the rats administered the extract had an average GLP-1 level of above 600 ng/L (Figure 4).

The highest GLP-1 levels (1022.918 ng/L) were observed in the group administered 500 mg/kg BW extract (*p* < 0.05). The low levels of GLP-1 observed in the groups that did not receive the extract were consistent with the increase in the levels of DPP-4, which degraded GLP-1.

**Figure 5.**
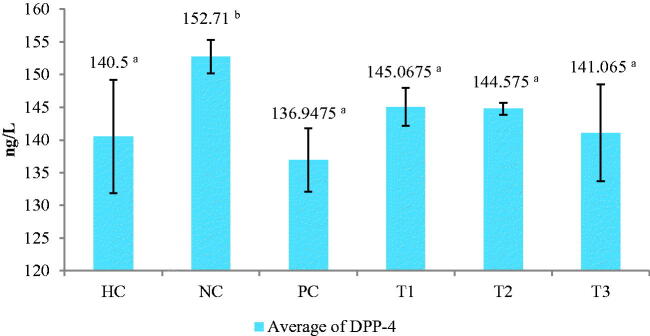
Dipeptidyl peptidase (DPP)-4 levels after extract treatment. Histograms with the same letter are not significantly different, values are expressed as mean ± SD (*n*: 24) *p* < 0.05. HC: Healthy control; NC: negative control (45 mg STZ); PC: positive control (45 mg metformin); T1: 250 mg ethanol extract of *C. comatus*; T2: 500 mg ethanol extract of *C. comatus*; T3: 750 mg ethanol extract of *C. comatus*.

The DPP-4 enzyme levels are significantly different in the NC group (*p* < 0.05) increased above 150 ng/L; whereas, in the groups treated with the extract, the DPP-4 levels were below 150 ng/L. The lowest DPP-4 levels were observed in the group treated with 750 mg/kg BW extract (Figure 5). High DPP-4 enzyme levels were closely correlated with low insulin and GLP-1 hormone levels. The decrease in DPP-4 levels could be associated with the bioactive compounds present in the *C. comatus* ethanol extract, such as comatins, and potential antioxidant compounds like rutin and vitamins E and C.

The lowest GSH level, significantly different in the NC rat group (*p* < 0.05), was below 3 µmol/L (Table 5). Low levels of GSH can occur due to increase in blood glucose levels, which in turn can increase NEG reactions that lead to increased aldehyde toxicity of protein amine groups and reduce GSH synthesis.

**Table 5.** Average of glutathione (GSH) levels.

Treatment group	Glutathione level (µmol/L)
HC	5.45 ± 0.560^b^
NC/IC	2.8 ± 0.565^a^
PC	4.225 ± 1.302^b^
T1	3.075 ± 0.050^a^
T2	3.153 ± 0.242^a^
T3	3.015 ± 0.017^a^

*Note*: Numbers on each line followed by the same letter are not significantly different, values are expressed as mean ± SD (*n*: 24) *p* < 0.05. HC: Healthy control; NC: negative control (45 mg STZ); PC: positive control (45 mg metformin); T1: 250 mg ethanol extract of *C. comatus*; T2: 500 mg ethanol extract of *C. comatus*; T3: 750 mg ethanol extract of *C. comatus*.

In addition, low levels of GSH have implications for defense against free radicals in pancreatic β-cell. The GSH levels (*p* < 0.05) in the experimental groups of rats treated with the *C. comatus* ethanol extract showed a good response, with GSH levels above 3 µmol/L; the highest level was obtained in the group treated with 500 mg/kg BW extract.

The BW of the rats in all experimental groups increased after administration of the *C. comatus* ethanol extract. The most significant increase in BW occurred in the group treated with 250 mg/kg BW extract (*p* < 0.05), which showed up to 10.07% steady increase in BW when compared to the NC group. The NC group rats experienced an average weight loss of 1.34% (Table 6).

**Table 6.** Average of increasing of body weight.

Treatment group	Initial body weight (g)	Late body weight (g)	Increasing body weight (%)
HC	110.75 ± 8.301^a^	132 ± 18.384^a^	19.19
NC/IC	149 ± 8.286 d^bc^	147 ± 15.811^ab^	−1.34
PC	136.5 ± 4.123^b^	139.25 ± 20.155^ab^	2.01
T1	144 ± 16.268^bc^	158.5 ± 21.946^ab^	10.07
T2	182.25 ± 22.983^d^	199.75 ± 30.717^c^	9.60
T3	164 ± 20.116^cd^	166.5 ± 11.958^b^	1.52

*Note*: Numbers on each line followed by the same letter are not significantly different, values are expressed as mean ± SD (*n*: 24) *p* < 0.05. HC: Healthy control; NC: negative control (45 mg STZ); PC: positive control (45 mg metformin); T1: 250 mg ethanol extract of *C. comatus*; T2: 500 mg ethanol extract of *C. comatus*; T3: 750 mg ethanol extract of *C. comatus*.

**Figure 6.**
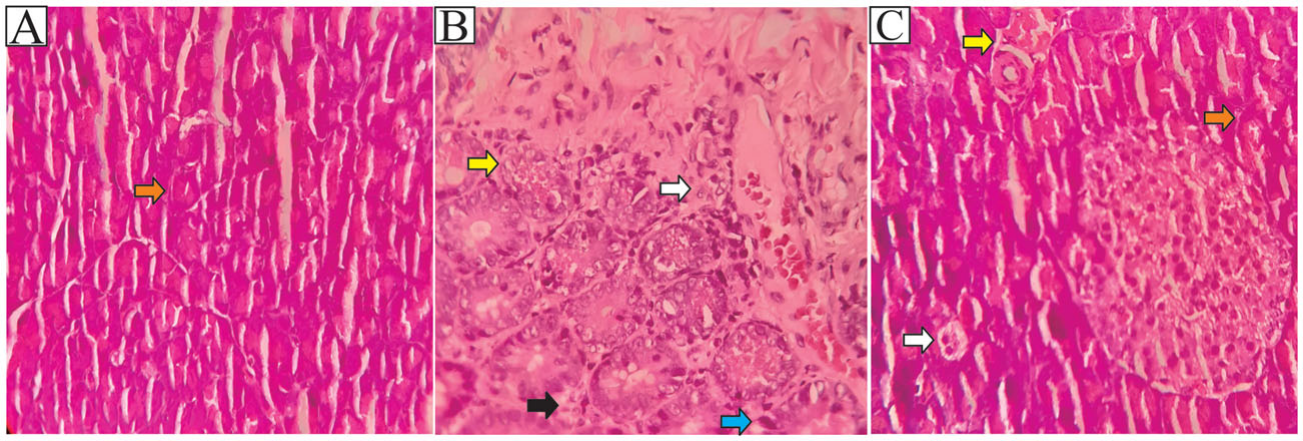
Histological effects in pancreas after treatment of HC, NC and PC group. HC (healthy control) (A), NC (negative control – STZ induction) (B), PC (positive control – given metformin) (C), normal cells (orange arrow), necrosis cells (yellow arrow), inflammatory cells (white arrow), pyknotic cells (blue arrow), and karyolytic cells (black arrow). Magnification 400×.

The histological observations result of pancreatic tissue in the HC group showed that the cells were in good condition and no abnormalities were found (Figure 6(A)). In this study, histological observations of the pancreas after STZ induction dose of 45 mg showed a significant effect on Langerhans islet cells (Figure 6(B)). In the NC group, cells experienced necrosis, inflammation, pyknosis, and karyolysis, with cell wall damage and lysis. Meanwhile, in the PC group that was given 45 mg metformin, the normal cells were more abundant than the necrotic and inflammatory cells (Figure 6(C)).

**Figure 7.**
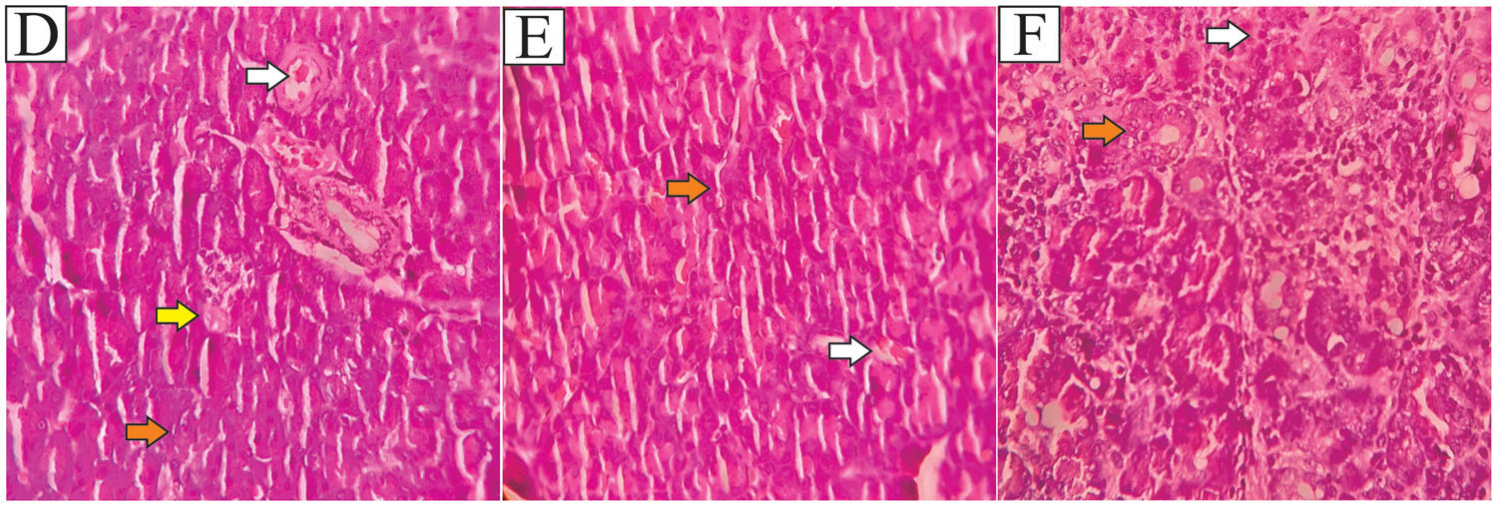
Histological effects in pancreas after treatment of T1, T2 and T3 group. Histological effects in pancreas after treatment. T1 (given of fruit body ethanol extract of *C. comatus* 250 mg) (D), T2 (given of fruit body ethanol extract of *C. comatus* 500 mg) (E), T3 (given of fruit body ethanol extract of *C. comatus* 750 mg) (F), normal cells (orange arrow), necrosis cells (yellow arrow), and inflammatory cells (white arrow). Magnification 400×.

The observations result of the PC group showed a few parts that were inflamed and necrosis. While the T1, T2, and T3 groups the number of normal or healthy cells appeared to be higher than in the NC group (Figure 7).

Different results were also shown in the group of rats were given the ethanol extract of *C. comatus* (Figure 7), the T1 group showed the condition of Langerhans islet cells that appeared to have little necrosis and inflammation (Figure 7(D)), while the T2 and T3 group showed different results were normal cell conditions more than in the T1 group, and little of necrosis and inflammatory cells (Figure 7(E,F)). These results indicate that the administration of *C. comatus* ethanol extract has a preventive and protective effect on Langerhans islet cells and minimises the pathogenicity that occurs in DM conditions.

## Discussion

The potential of bioactive compounds such as flavonoid, alkaloid, and saponin as anti-diabetic and rutin, as well as vitamins C and E as antioxidant agents in ethanol extracts of *C. comatus,* was investigated using qualitative and quantitative analysis of HPLC results to identify the type and quantity of these compounds. In this study, the following bioactive compounds were identified from the *C. comatus* ethanol extract: flavonoids, alkaloids, saponins, vitamin E, vitamin C, quercetin, and rutin. From these, flavonoids, vitamin C, and vitamin E are known to have antioxidant activity and extremely potent reducing effects on free radicals formed in DM (Atanassova et al. 2011). The antioxidant ability of extracts from the fruiting body and submerged *C. comatus* cultures could be attributed to the polyphenol and flavonoids that help to stabilise free radicals by donating hydrogen ions (H^+^) to free radicals (Tešanović et al. 2017).

In this study, STZ 45 mg was induced to act as a diabetogenic agent via pancreatic β-cell destruction. Blood glucose levels were increased to >150 mg/dL following STZ induction. STZ induction has been shown to result in a > 200 mg/dL increase in fasting blood glucose levels (Gajdošík et al. 1999). STZ was incorporated into the pancreatic β-cell via the glucose transporter GLUT2. STZ inhibits glucose oxidation and inhibits the synthesis and secretion of insulin. Streptozotocin’s action on pancreatic β-cell is accompanied by changes in distinctive blood sugar and insulin levels. Given that STZ is a donor of nitric oxide (NO) and that NO has been shown to destroy pancreatic islet cells, it has been suggested that this molecule contributes to STZ-induced DNA damage, DNA alkylation, guanylyl cyclase activity, and increased cGMP formation. Pancreatic β-cells have significantly lower endogenous antioxidant levels than cells from other organs in the body and are much more susceptible to oxidative damage caused by reactive oxygen species (ROS). STZ has been shown to inhibit the Krebs cycle and significantly decrease mitochondrial oxygen consumption. These effects significantly reduce mitochondrial ATP production and result in depletion of this nucleotide in the β-cell. As a result of the cellular NAD^+^ and ATP depletion, inhibition of insulin synthesis and secretion will be reduced (Sakurai et al. 2001; Szkudelski 2001).

**Figure 8.**
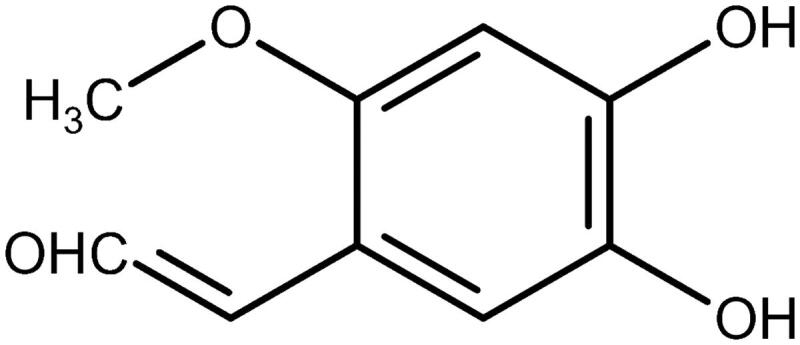
Structure of comatin (4,5-dihydroxy-2-methoxy-benzaldehyde).

A decrease in blood glucose levels after administering the ethanol extract dose of 750 mg/kg BW exhibited a better effect than the dose of 250 mg/kg BW and 500 mg/kg BW (Table 3). The decrease in blood glucose was caused by the inhibition of α-glucosidase activity by rutin and comatin in the *C. comatus* extracts. Rutin inhibits the expression of the glucose transporter (GLUT) 2 in the intestinal mucosa, resulting in decreased glucose and fructose absorption in the intestine, lowering blood glucose levels. According to Ding et al. (2010), comatin obtained from 80 mg/kg *C. comatus* mycelia submerged fermentation could lower blood glucose levels in diabetic rats from 5.14 to 4.28 mM. Comatin’s anti-diabetic activity results from its chemical structure, which contains hydroxyl groups at positions 4 and 5 (Figure 8).

Inhibition of the tricarboxylic acid cycle and glycolytic pathways can result in elevated serum glucose levels, whereas increased NEG reactions can result in elevated blood glucose levels (Zhang and Cui 2007). Comatin inhibits NEG in a manner similar to rutin; thus, inhibition of hexokinase and pyruvate dehydrogenase can be reversed, and glycolysis can resume normal function, resulting in decreased blood glucose levels. Rutin and its metabolites have been shown to inhibit the initial reaction in the formation of NEG and AGEs (Cervantes-Laurean et al. 2006). The ethanol extract of *C. comatus* was found to contain a high concentration of rutin and flavonoids in this study. In a previous study, administration of flavonoid compounds such as rutin, quercetin, boswellic acid, and ellagic acid to rats at 100 mg significantly decreased glucose and triglyceride levels compared to the control, an effect attributed to flavonoid activity. Rutin has a greater ability to lower blood glucose levels than other flavonoids. This decrease results from decreased hepatic sugar consumption and inhibition of glucose transport in the intestine (Jadhav and Puchchakayala 2012).

A decrease in HbA1c levels after administering ethanol extract dose of 500 mg/kg BW exhibited the best reduction effect of HbA1c levels than the dose of 250 and 750 mg/kg BW (Table 4). HbA1c levels decrease in lockstep with blood glucose levels, while increasing HbA1c levels result in an increase in blood glucose levels. Blood cell glycosylation has been attributed to a more rapid and significant rise in blood glucose than insulin levels (Ghezeljeh et al. 2017). A rise in HbA1c levels of more than 8–8.5% during fasting is intolerable in humans. Typically, red blood cells undergo glycosylation reactions, but these reactions occur slowly and continuously over the 120-day lifetime of a red blood cell in the DM state. When blood glucose levels are elevated, the glycosylation reaction is accelerated and results in a transition from chronic to acute state when HbA1c levels are greater than 10% in the fasting state (Davidson et al. 2005). Previous research has demonstrated that administering an ethanol extract of *C. comatus* to diabetic rats can reduce HbA1c levels by 6.35% at a dose of 500 mg extract (Ratnaningtyas et al. 2019). Additionally, 250 mg of ethyl acetate extract of *C. comatus* reduced HbA1c levels by 3.77% (Husen et al. 2021). Therefore, this study demonstrated that the ethanol extract of *C. comatus* can decrease HbA1c levels by 4–4.3%. Thus, the hypothesis that *C. comatus* can reduce HbA1c levels in DM rats is strengthened.

This study discovered an increase in insulin levels following treatment. The administration of 250 mg/kg BW of *C. comatus* ethanol extract resulted in the most significant increase in insulin levels compared to 500 and 750 mg/kg BW (Figure 3). Increased antioxidant defense in the pancreatic β-cell enables uninterrupted metabolism and insulin production. Vitamin E is a fat-soluble nutrient critical for protecting β-cell membranes from free radical attack (Niki 2014). Vitamin E is a powerful antioxidant that neutralises active free radicals by transferring hydrogen atoms to form non-radical products (Ratnaningtyas et al. 2021). Vitamin C is a water-soluble antioxidant that acts as a free radical scavenger. Vitamins C and E work synergistically to protect pancreatic β-cells and enable normal insulin production (Olson and Seidel 2000). Previously published research demonstrated that administering a 250 mg dose of *C. comatus* ethyl acetate extract increased insulin levels by 8.11 mIU/mL. (Husen et al. 2021). Comatin from *C. comatus* inhibits phosphodiesterase and increases cAMP levels in pancreatic β-cells, which stimulates the release of protein kinase A (PKA) and increases insulin secretion (Donath et al. 2008).

Additionally, this study demonstrated that the administration of ethanol extract at a dose of 500 mg/kg BW had a more significant effect on increasing GLP-1 levels than 250 and 750 mg/kg BW. Compared to the NC group, the 500 mg dose increased GLP-1 levels by 71.09% (Figure 2). Previously published research demonstrated that administration of a 500 mg dose of *C. comatus* ethyl acetate extract increased GLP-1 levels by 61.77% compared to the NC group (Husen et al. 2021). NEG, which occurs due to increased free radicals and pancreatic β-cell destruction, results in decreased GLP-1. The GLP-1 receptor (GLP-1R) in pancreatic β-cell is unable to bind to GLP-1 due to lipid peroxidation in the β-cell membranes, which results in increased intracellular (DNA) damage and ultimately in the onset of apoptosis or cellular senescence (Oeseburg et al. 2010), resulting in a significant drop in insulin levels in NC rats induced with STZ and given no treatment due to a The DM model increases the activity of the DPP-4 enzyme, which degrades GLP-1 over time (Drucker 2007). The alkaloids and comatin in the *C. comatus* ethyl acetate extract acted as DPP-4 inhibitors, preventing the DPP-4 enzyme from degrading GLP-1 (Husen et al. 2021).

Decreasing DPP-4 levels correspond with the increase of GLP-1 levels, and the administration of ethanol extract dose of 750 mg/kg BW exhibited the best decreasing effect of DPP-4 levels than the dose of 250 and 750 mg/kg BW (Figure 3). Protection of pancreatic β-cell from lipid peroxidation reactions by antioxidant compounds can lead to the optimisation of insulin formation through the inhibition of DPP-4 activity by comatin, which supports the binding of GLP-1 with its receptors and subsequent insulin secretion. Targeted inactivation of the DPP-4 gene in rats also leads to increased plasma levels of GIP, GLP-1, and insulin and a reduced glycemic excursion following oral glucose challenge, DPP‐4 inhibition represents another potential strategy to increase plasma concentration of GLP‐1 to enhance the incretin effect (Drucker 2007; Tasyurek et al. 2014).

The administration of 500 mg/kg BW ethanol extract had the greatest effect on increasing GLP-1 levels compared to 250 and 750 mg/kg BW ethanol extract (Table 5). Certain amino acids found in *C. comatus*, such as methionine, are required to form GSH. Additionally, the ethanol extract of *C. comatus* contains antioxidants such as rutin, vitamin E, and vitamin C, which acts as an exogenous antioxidant to protect against free radical attack and minimise sulfhydryl group oxidation in GSH (Buettner 1993). Increased free radicals reduce GSH synthesis levels because they can easily attack the sulfhydryl groups (–SH) of GSH (Kyseĺová et al. 2005). GSH is an endogenous antioxidant present in the body that prevents the formation of H_2_O_2_ and functions as a co-substrate during the formation of glutathione peroxidase (GPx). GPx is essential for the decomposition of H_2_O_2_ into water (H_2_O) and oxygen (O_2_) (Winarsi et al. 2012).

Histology of the Islets of Langerhans is necessary to ascertain the pancreas’s health status as an organ that secretes critical hormones that control blood glucose levels. This study indicated that administering *C. comatus* ethanol extract resulted in a reduction in inflamed and necrotic cells compared to the NC group. Cell damage in the Islets of Langerhans can occur for a variety of reasons, including the induction of STZ, which enters pancreatic cells directly via the GLUT-2 pathway, or as a result of reactive oxygen species (ROS) that cause oxidative stress. The increase in free radicals caused by STZ induction is due to an increase in NO production, which increases the activity of the xanthine oxidase enzyme (XOD). Increased levels of the XOD enzyme result in inflammation and cell damage (Szkudelski 2001). Previous research showed that induction of alloxan 100 mg/kg increased XOD activity by 2.337 ± 0.31 nmol/mL, while normal rats had XOD activity of 1.958 ± 0.181 nmol/mL, and the group with *C. comatus* suspension showed XOD activity of 2.097 ± 0.294 nmol/mL (Popovic et al. 2001). A previous study by inducing STZ at a dose of 50 mg/kg BW showed that the average number of cells in the Islets of Langerhans was 37.25 ± 3.41, while after administration of exopolysaccharide (EPS) *C. comatus* was 277.50 ± 19.44. The mean area of ​​the Islets of Langerhans in the NC group was also low, with 5271.25 ± 113.89 nm^2^, while the average EPS group was 40424.50 ± 393.50 (Yamac et al. 2009). In this study, it was shown that the antioxidant activity of *C. comatus* prevent oxidative stress and lipid peroxidation reactions in pancreatic β-cell; preventing necrosis, karyolysis, pyknosis and inflammation due to increased free radicals that generated by STZ. Vitamins C, E, rutin, and flavonoids play an important role as free radical scavengers.

In this study, the dose of 250, 500, and 750 mg/kg BW can increase BW levels, while the NC group experienced weight loss (Table 6). Although they are not definite indications of acute DM, hyperglycaemia conditions and weight loss are often observed in DM. Drastic changes in BW from either over-eating (polyphagia) or over-drinking (polydipsia) can be used as a DM acute indication. The nutritional status and ideal BW for an individual should be considered when analysing changes in BW that occur following anti-diabetic agent administration (Fatimah 2015). The results showed that the average BW of ethanol extract administered rats was 155–200 g and was consistent with previous findings in normal Wistar rats (2–3 months old), which have an average BW of 150–200 g under normoglycemic conditions (Murwani et al. 2006). Previous research in 100 mg of alloxan-induced DM rats model results in blood glucose levels of >250 mg/dL. Increased blood glucose levels affect body weight loss by >12%. The loss of BW in DM rats is caused by the decreasing rate of glucose catabolism and biosynthesis of fat and protein (Obasi et al. 2019). Meanwhile, a study revealed that when DM rats were induced with a 55 mg dose of STZ, blood glucose levels increased to >350 mg/dL, and HbA1c levels increased to 12.52% on day 28, resulting in a 28.33% weight loss. BW loss occurs due to excessive tissue protein breakdown and uncontrolled glycaemic conditions (Gandhi and Sasikumar 2012). In diabetes mellitus, pancreatic-cell destruction can result in fatigue and weight loss (Dwikayana et al. 2016). Modelling HbA1c weight, insulin, and glucose (WHIG) levels in type 2 diabetes mellitus patients demonstrated that weight changes could occur due to HbA1c, insulin, and glucose levels (Choy et al. 2016).

This study demonstrated that extract of *C. comatus* lowers blood glucose by increasing plasma insulin levels via increased GLP-1 and inhibition of DPP-4. This has been confirmed in previous studies where *C. comatus* and its comatin could inhibit DPP-4 activity, reduce free radicals, and increase GLP-1 levels (Ding et al. 2010; Husen et al. 2021; Ratnaningtyas et al. 2021). As a result, it is strongly suggested that *C. comatus* protects the pancreas, lowers blood glucose, and increases insulin and GLP-1 levels in STZ-induced diabetes mellitus rats by inhibiting DPP-4 and attenuating ROS.

### Limitations and further research

This analysis of the bioactive compounds in the *C. comatus* ethanol extract was limited to qualitative and quantitative tests involving spectrophotometry and HPLC. Qualitative analysis is limited to general content information and is not specific to certain compounds, so that information on a single bioactive compound cannot be identified in detail, besides qualitative identification does not provide information about the amount or content of the compounds that present in the extract. Whereas the limitation of using spectrophotometry is that it only provides the information related to the bioactive compounds at the group level in total and cannot measure in detail every type of its group. While the limitations of using HPLC are the availability of standard compounds to be used and the types of HPLC columns that suitable for analysis of these compounds. Diethyl ether was used for experimental anaesthesia according to the guidelines of the ethics committee, and we recommend the use of other anaesthetic compounds such as isoflurane, which has faster anaesthetic activity and response and reduces suffering and pain in animals. Another limitation is the evaluation of IC_50_ for the *C. comatus* fruit body ethanol extract; moreover, the LD_50_ for *C. comatus* fruit body against the experimental rat groups have not yet been defined. Further analysis of the fractionation of the ethanol extract from the *C. comatus* fruiting body is also needed to obtain a information about the specific single compounds that can be used as an antioxidant and anti-diabetic agents. Further investigations using high-resolution mass spectrometry (HR-MS) and Fourier-transform infra-red spectroscopy (FTIR) would aid in obtaining comatin. Fractionation with gas chromatography–mass spectrometry could provide a more in-depth analysis of the compounds present in the effervescent such as flavonoid, alkaloids or vitamins that contained in *C. comatus* and to analyse its safety when consumed as a herbal medicine to minimise the adverse effects on the liver. These analyses should include measurement of the aspartate transaminase and alanine aminotransferase levels present in *C. comatus* extracts. Additionally, the effect of antioxidants on DM should be further evaluated using electron microscopy to visualise the histological attributes of pancreatic β-cell. Preliminary preclinical tests can be conducted on the effervescent products that have been tested and evaluated for safety in experimental animal models. The findings of these preclinical tests can be applied to patients who have type 2 DM and are not dependent on insulin as a therapeutic companion drug, to find more biological effects of *C. comatus*.

## Conclusions

This study found that DPP-4 and GLP-1 are important parameters in the evaluation of DM severity. The degradation of GLP-1 in DM occurred due to DPP-4 activity, which caused the reduction in insulinotropic activity of GLP-1. Qualitative and quantitative analysis showed the presence of bioactive compounds present in *C. comatus* mushroom extracts that were effective in reducing HbA1c levels and increasing GLP-1 and GSH levels. The extract could reduce blood glucose and DPP-4 enzyme levels as well as increase insulin hormone levels in a dose-sensitive manner. The dose of 750 mg/kg exhibited the best effects in lowering blood glucose levels and the DPP-4 enzyme. The dose of 500 mg/kg exhibited the best effects in lowering HbA1c levels and increasing GLP-1 and GSH levels, and the dose of 250 mg/kg exhibited the best effect in increasing insulin levels. Our results indicated that *C. comatus* can be developed into herbal medicine for DM treatments that make use of its bioactive compounds that act as DPP-4 inhibitors and increase the insulinotropic activity of GLP-1. Further in-depth studies will be required to identify the bioactive compounds in *C. comatus*; fractionation of these compounds would be useful for the further analysis of their biological responses or effects. Future studies of additional biochemical, physiological, and molecular parameters could confirm the effects of the *C. comatus* ethanol extract on the metabolic processes that contribute to the extremely complex conditions in DM.

## Supplementary Material

Supplemental Material

Supplemental Material

## Data Availability

The data supporting the findings of this study are available within the article and its supplementary materials.
