# Supplementary material for: Ethanol extract of the mushroom Coprinus comatus exhibits antidiabetic and antioxidant activities in streptozotocin-induced diabetic rats
Source: Pharm Biol. 2022 Jun 8;60(1):1126–36. doi: 10.1080/13880209.2022.2074054 (PMC9186368; doi:10.1080/13880209.2022.2074054)
Supplement: Supplemental Material [file IPHB_A_2074054_SM5874.zip › Rutin_Result_Analysis_Ethanol_Extract.pdf]

SHIMADZU LabSolutions

Analysis Report

<Sample Information>

Sample Name	: Ekstrak etanol jamur Coprinus C		
Sample ID	:		
Data Filename	: Ekstrak etanol jamur Coprinus C.lcd		
Method Filename	: rutin.lcm		
Batch Filename	:		
Vial #	: 1-1	Sample Type	: Unknown
Injection Volume	: 7 uL		
Date Acquired	: 12/09/2019 2:49:55 PM	Acquired by	: System Administrator
Date Processed	: 12/09/2019 3:18:14 PM	Processed by	: System Administrator

<Chromatogram>

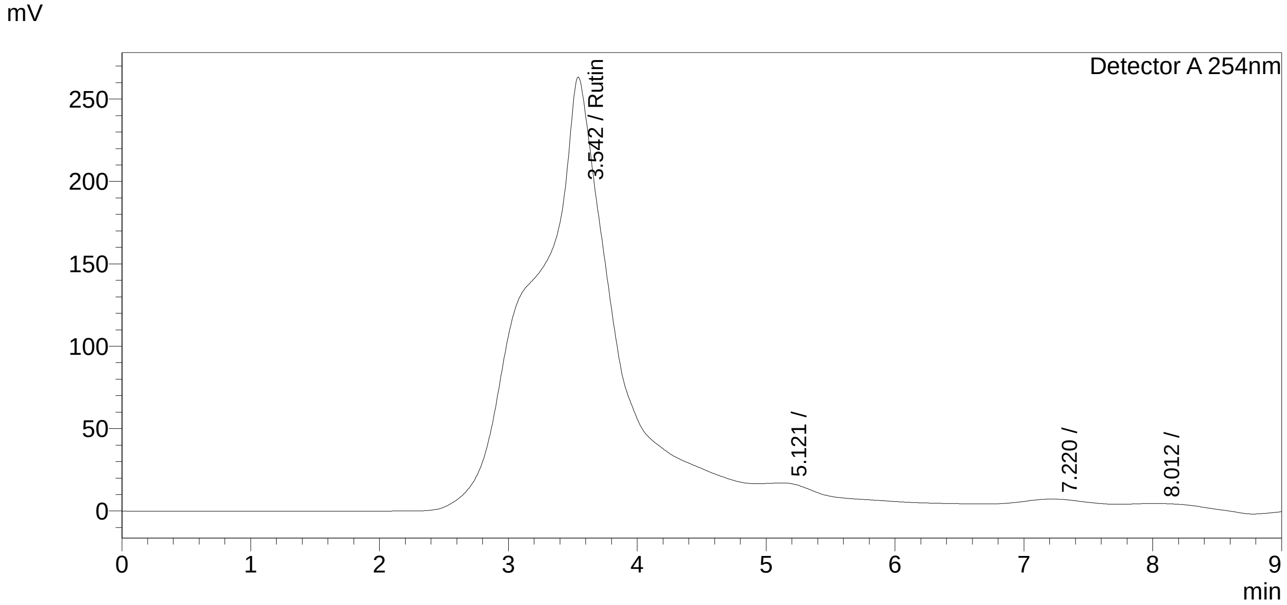

<Peak Table>

Detector A 254nm

Peak#	Ret. Time	Area	Height	Conc.	Unit	Mark	Name
1	3.542	13304778	263876	495.608	ppm	S	Rutin
2	5.121	64023	3103	0.000		T	
3	7.220	177930	4524	0.000		T	
4	8.012	179700	4208	0.000		TV	
Total		13726431	275711				
