# Supplementary material for: Ethanol extract of the mushroom Coprinus comatus exhibits antidiabetic and antioxidant activities in streptozotocin-induced diabetic rats
Source: Pharm Biol. 2022 Jun 8;60(1):1126–36. doi: 10.1080/13880209.2022.2074054 (PMC9186368; doi:10.1080/13880209.2022.2074054)
Supplement: Supplemental Material [file IPHB_A_2074054_SM5874.zip › Vitamin_C_Result_Analysis_Ethanol_Extract.pdf]

Analysis Report

<Sample Information>

Sample Name	: Ekstrak etanol jamur Coprinus C		
Sample ID	:		
Data Filename	: Ekstrak etanol jamur Coprinus C.lcd		
Method Filename	: Asam Askorbat - Copy.lcm		
Batch Filename	:		
Vial #	: 1-1	Sample Type	: Unknown
Injection Volume	: 20 uL		
Date Acquired	: 12/09/2019 11:59:12 AM	Acquired by	: System Administrator
Date Processed	: 12/09/2019 12:07:52 PM	Processed by	: System Administrator

<Chromatogram>

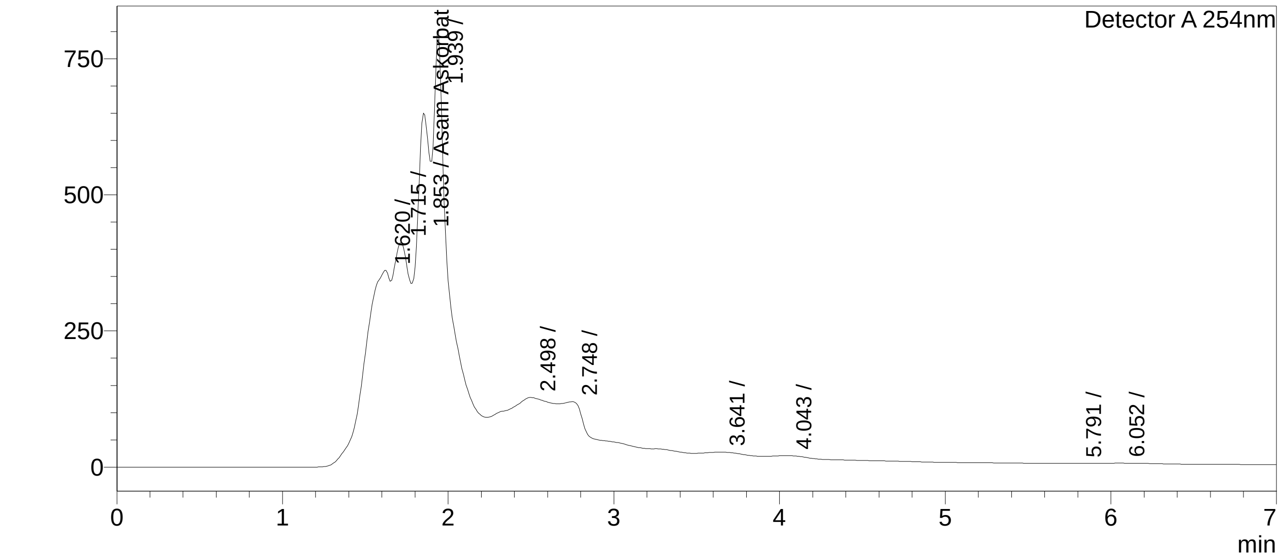

<Peak Table>

Detector A 254nm

Peak#	Ret. Time	Area	Height	Conc.	Unit	Mark	Name
1	1.620	3740969	361134	0.000			
2	1.715	2829499	412239	0.000		V	
3	1.853	3941327	649591	152.106	mg/L	V	Asam Askorbat
4	1.939	5840501	801367	0.000		V	
5	2.498	2865495	126706	0.000		V	
6	2.748	2545409	118766	0.000		V	
7	3.641	573653	25543	0.000		V	
8	4.043	875368	18614	0.000		V	
9	5.791	71987	3443	0.000		V	
10	6.052	91079	3260	0.000		V	
Total		23375287	2520664				
