# Supplementary material for: Ethanol extract of the mushroom Coprinus comatus exhibits antidiabetic and antioxidant activities in streptozotocin-induced diabetic rats
Source: Pharm Biol. 2022 Jun 8;60(1):1126–36. doi: 10.1080/13880209.2022.2074054 (PMC9186368; doi:10.1080/13880209.2022.2074054)
Supplement: Supplemental Material [file IPHB_A_2074054_SM5874.zip › Vitamin_E_Result_Analysis_Ethanol_Extract.pdf]

SHIMADZU LabSolutions

Analysis Report

<Sample Information>

Sample Name	: Ekstrak etanol jamur Coprinus C		
Sample ID	:		
Data Filename	: Ekstrak etanol jamur Coprinus C.lcd		
Method Filename	: vitamin E - Copy.lcm		
Batch Filename	:		
Vial #	: 1-1	Sample Type	: Unknown
Injection Volume	: 20 uL		
Date Acquired	: 12/09/2019 12:40:04 PM	Acquired by	: System Administrator
Date Processed	: 12/09/2019 12:50:43 PM	Processed by	: System Administrator

<Chromatogram>

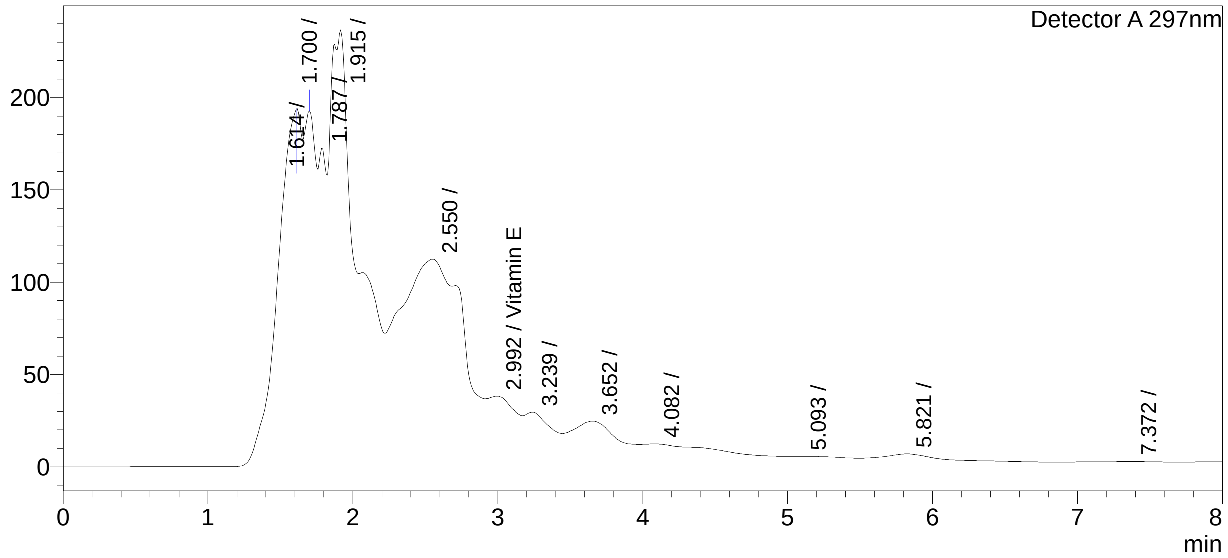

<Peak Table>

Detector A 297nm

Peak#	Ret. Time	Area	Height	Conc.	Unit	Mark	Name
1	1.614	2167720	193738	0.000			
2	1.700	1169918	192662	0.000		V	
3	1.787	661392	172170	0.000		V	
4	1.915	3270182	236124	0.000		V	
5	2.550	3552248	111900	0.000		V	
6	2.992	505449	37539	53.856	g/L	V	Vitamin E
7	3.239	391181	28771	0.000		V	
8	3.652	566465	23757	0.000		V	
9	4.082	472678	11223	0.000		V	
10	5.093	111973	4168	0.000		V	
11	5.821	179191	5170	0.000		V	
12	7.372	16773	416	0.000		V	
Total		13065172	1017638				
