# Supplementary material for: Ethanol extract of the mushroom Coprinus comatus exhibits antidiabetic and antioxidant activities in streptozotocin-induced diabetic rats
Source: Pharm Biol. 2022 Jun 8;60(1):1126–36. doi: 10.1080/13880209.2022.2074054 (PMC9186368; doi:10.1080/13880209.2022.2074054)
Supplement: Supplemental Material [file IPHB_A_2074054_SM5873.zip › Rutin_Result_Analysis_Standard_.pdf]

SHIMADZU LabSolutions

Analysis Report

<Sample Information>

Sample Name	: Rutin 100 ppm		
Sample ID	:		
Data Filename	: Rutin 100 ppm.lcd		
Method Filename	: rutin.lcm		
Batch Filename	:		
Vial #	: 1-1	Sample Type	: Standard
Injection Volume	: 7 uL	Level	: 1
Date Acquired	: 6/09/2019 1:12:22 PM	Acquired by	: System Administrator
Date Processed	: 6/09/2019 2:10:04 PM	Processed by	: System Administrator

<Chromatogram>

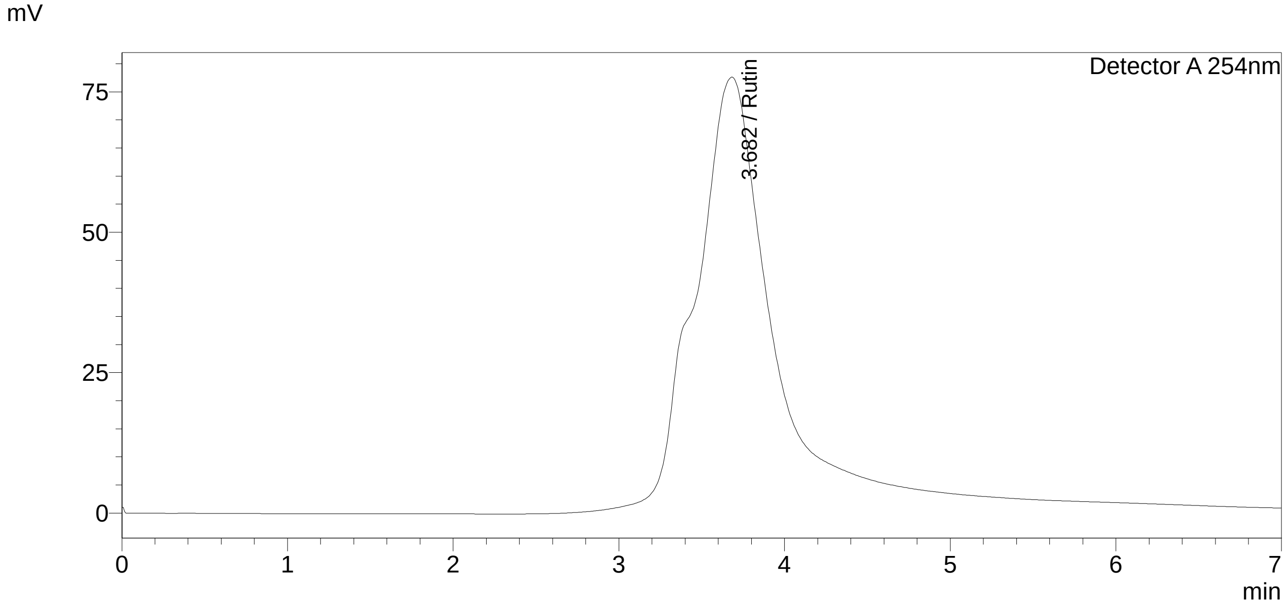

<Peak Table>

Detector A 254nm

Peak#	Ret. Time	Area	Height	Conc.	Unit	Mark	Name
1	3.682	2684538	77490	100.000	ppm		Rutin
Total		2684538	77490				
