# Supplementary material for: Ethanol extract of the mushroom Coprinus comatus exhibits antidiabetic and antioxidant activities in streptozotocin-induced diabetic rats
Source: Pharm Biol. 2022 Jun 8;60(1):1126–36. doi: 10.1080/13880209.2022.2074054 (PMC9186368; doi:10.1080/13880209.2022.2074054)
Supplement: Supplemental Material [file IPHB_A_2074054_SM5873.zip › Vitamin_C_Result_Analysis_Standard_.pdf]

SHIMADZU LabSolutions

Analysis Report

<Sample Information>

Sample Name : std vit C
Sample ID :
Data Filename : std vit C.lcd
Method Filename : Asam Askorbat - Copy.lcm
Batch Filename :
Vial # : 1-1
Injection Volume : 20 uL
Date Acquired : 25/06/2019 3:20:37 PM
Date Processed : 31/07/2019 11:34:44 AM

Sample Type : Standard
Level : 1
Acquired by : System Administrator
Processed by : System Administrator

<Chromatogram>

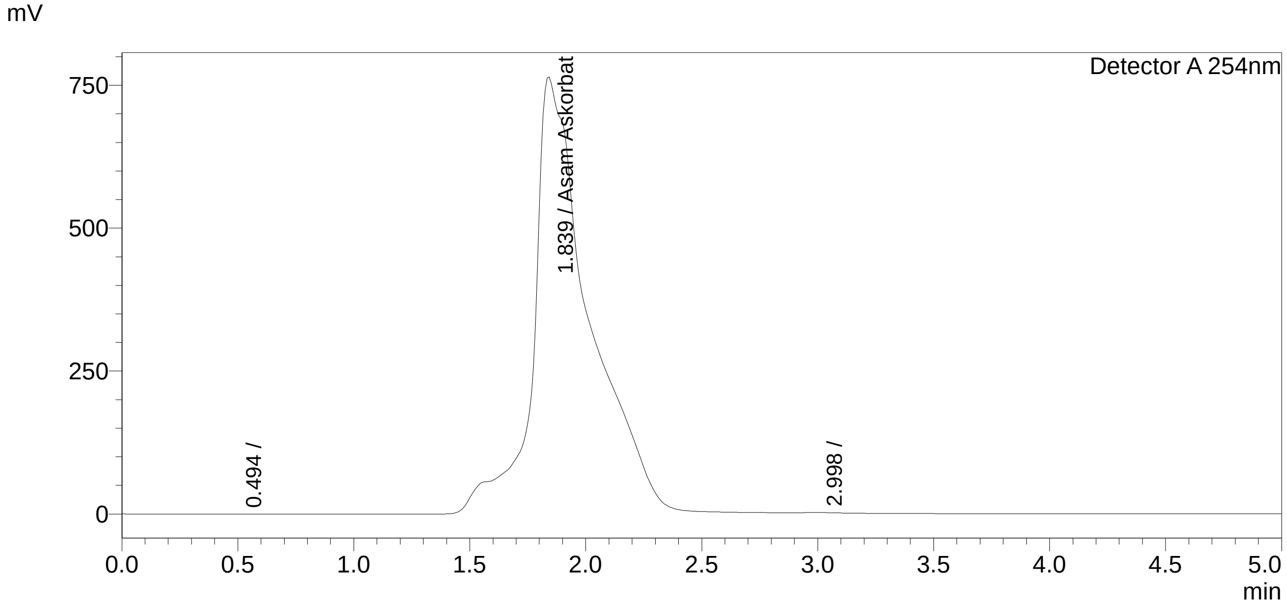

<Peak Table>

Detector A 254nm

Peak#	Ret. Time	Area	Height	Conc.	Unit	Mark	Name
1	0.494	2022	78	0.000			
2	1.839	12955870	764451	500.000	mg/L	S	Asam Askorbat
3	2.998	8982	831	0.000		T	
Total		12966873	765360				
