# Supplementary material for: Ethanol extract of the mushroom Coprinus comatus exhibits antidiabetic and antioxidant activities in streptozotocin-induced diabetic rats
Source: Pharm Biol. 2022 Jun 8;60(1):1126–36. doi: 10.1080/13880209.2022.2074054 (PMC9186368; doi:10.1080/13880209.2022.2074054)
Supplement: Supplemental Material [file IPHB_A_2074054_SM5873.zip › Vitamin_E_Result_Analysis_Standard_.pdf]

SHIMADZU LabSolutions

Analysis Report

<Sample Information>

Sample Name	: standart vit E		
Sample ID	:		
Data Filename	: standart vit E.lcd		
Method Filename	: vitamin E - Copy.lcm		
Batch Filename	:		
Vial #	: 1-2	Sample Type	: Standard
Injection Volume	: 20 uL	Level	: 1
Date Acquired	: 1/08/2019 11:35:53 AM	Acquired by	: System Administrator
Date Processed	: 1/08/2019 12:39:05 PM	Processed by	: System Administrator

<Chromatogram>

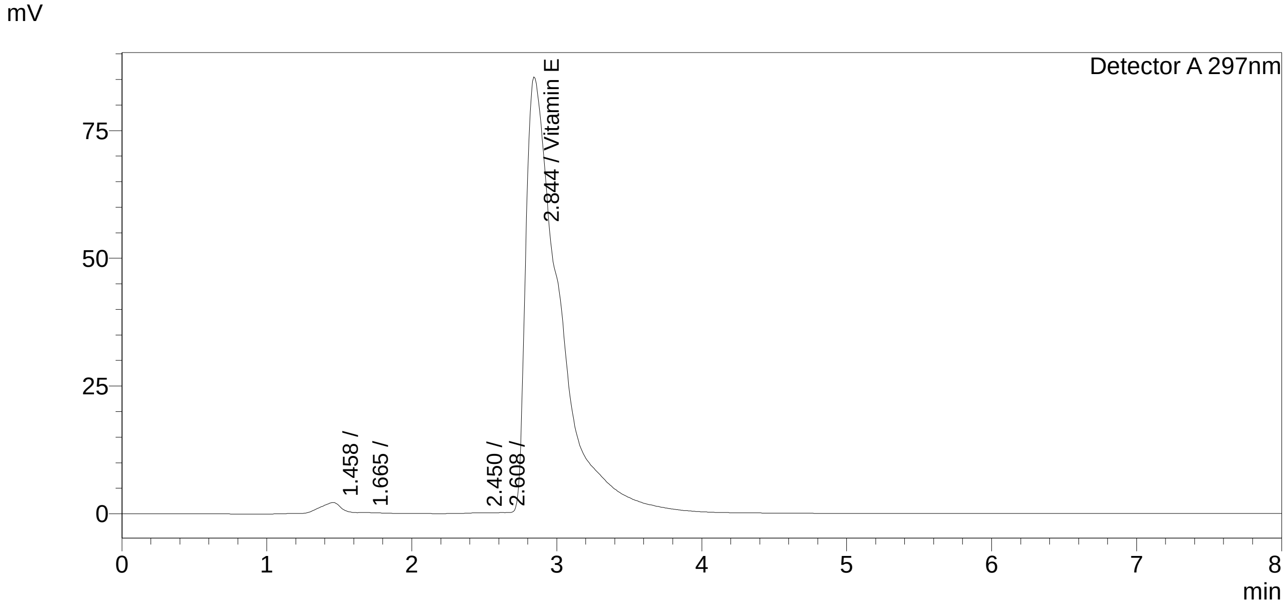

<Peak Table>

Detector A 297nm

Peak#	Ret. Time	Area	Height	Conc.	Unit	Mark	Name
1	1.458	24559	2236	0.000			
2	1.665	3506	259	0.000		V	
3	2.450	2120	181	0.000			
4	2.608	1642	214	0.000		V	
5	2.844	1407780	85470	150.000	g/L	V	Vitamin E
Total		1439607	88361				
